# 

**Published:** 1886-12

**Authors:** 


					﻿EiJiK t at i?ir	. nt Awtin !• m.«. c■ S><-- rM'J«« Mau
Vot II.
DECEMBER, x886.
W 6.
Tt ÄISTTTvT
aSWf£ ¿8*ikt jL irt JLwSi «Aaa^ Ä*A-
« » ri A11* ••!
MEfiW
|"| H w1
«
iinnuft»
d s Hv fcUfi-
/•	Ira ^-1
'■£> - /wwa'JI
F E. DANIEL, M D.. Ediior and Publisher, AUSTIN, TEXAS
Virihmi iiiliei ret7 l-ilbct-i	IMttladHpMa.
Eigene Von B<»i > km\xn, Sn im Book imiJoii Pkinthr, Ahiix, Ii.xu.
				

